# Impact on Product Appeal of Labeling Wine and Beer With (a) Lower Strength Alcohol Verbal Descriptors and (b) Percent Alcohol by Volume (%ABV): An Experimental Study

**DOI:** 10.1037/adb0000376

**Published:** 2018-08-30

**Authors:** Milica Vasiljevic, Dominique-Laurent Couturier, Theresa M. Marteau

**Affiliations:** 1 Behaviour and Health Research Unit, University of Cambridge

**Keywords:** alcohol policy, appeal, lower strength alcohol labeling, perceived strength, population health

## Abstract

Lower strength alcohol products may help reduce alcohol consumption and associated harms. This study assessed the impact of labeling wine and beer with different verbal descriptors denoting lower strength, with and without percent alcohol by volume (%ABV), on product appeal and understanding of strength. Three thousand three hundred ninety adult survey-panel members were randomized to 1 of 18 groups with 1 of 3 levels of verbal descriptor (*Low* vs. *Super Low* vs. *No verbal descriptor*) and 6 levels of %ABV (5 levels varying for wine and beer, and no level given). Products with verbal descriptors denoting lower strength (*Low* and *Super Low*) had lower appeal than *Regular* strength products. Appeal decreased as %ABV decreased. Understanding of strength was generally high across the various drinks with majority of participants correctly identifying or erring on the side of caution when estimating the units and calories in a given drink, appropriateness for consumption by children, and drinking within the driving limit. We discuss the theoretical and policy implications of these findings for public health.

Alcohol is the fifth leading cause of death and disability globally (Sassi, 2015). The development, promotion, and marketing of lower strength alcohol products (i.e., products containing lower than average alcohol by volume [ABV]) has been proposed as a viable strategy to reduce alcohol consumption. In the U.K. this is reflected in the most recent Government Alcohol Strategy published in March, 2012, which included an industry pledge through the Responsibility Deal to take one billion units out of the market by 2015, primarily through increasing consumer selection of lower strength alcohol products (Department of Health, 2012).

One way in which consumer selection of lower strength alcohol products could be encouraged further is by making the alcohol content of these products more explicit through labeling. Low strength alcohol labels are a set of labels that carry descriptors such as “low” or “lighter” to denote low or reduced alcohol content in alcohol beverages. Current legislation across the European Union (EU) limits the number of terms that can be used and further restricts the use of such descriptors to drinks of 1.2% ABV and lower (with similar restrictions found globally; see Canadian Food Inspection Agency, 2017; The European Parliament and the Council of the European Union, 2011; Food Standards Australia & New Zealand, 2014).

The U.K. national regulations covering the use of lower strength alcohol terms were repealed at the end of December 2014, with a sunset clause in place until the end of 2018. This provides an opportunity to consider revisions to the legislation to allow industry to use a wider variety of verbal descriptors denoting low/er alcohol strength and to extend the strength limit to include products lower than the current average on the market but higher than the current legislated cap of 1.2%ABV. Even though sales data show that the preferred products of choice are drinks of regular (average) strength (Department of Health, 2014), recent years have seen a growing interest from consumers in lower strength and no-alcohol products (see “Big brewers see strong potential for weak beer,” 2016; Wine Intelligence, 2013).

The potential of lower strength alcohols to reduce alcohol consumption at the population level depends on a number of assumptions: first, lower strength alcohols being selected instead of higher strength alcohols as opposed to simply increasing the number of occasions perceived suitable for consuming alcohol (for a discussion of this issue see Anderson & Rehm, 2016; Rehm, Lachenmeier, Llopis, Imtiaz, & Anderson, 2016; Vasiljevic, Coulter, Petticrew, & Marteau, 2018); and second, labels highlighting low/er alcohol strength not engendering a self-licensing effect (i.e., giving people permission to act indulgently following what might be interpreted as a virtuous choice) such that people overconsume lower strength alcohol products resulting in consumption of more units than would have been consumed from a higher strength product alone (see Khan & Dhar, 2006).

Although empirical evidence regarding these two assumptions is limited, a recent systematic review summarizing studies of product labeling denoting low fat, calorie, or salt content in food (*k* = 19) and low tar, mild/light flavor in tobacco (*k* = 6), suggested that such labels may encourage consumption of the labeled products by altering people’s perceptions concerning the content of products, and what they judge to be an appropriate serving (Shemilt, Hendry, & Marteau, 2017). The review did not identify any studies examining the effects of lower strength alcohol labeling.

Evaluations of other types of alcohol labeling may provide useful indications as to how the public might respond to lower strength alcohol labeling. For example, studies to date have shown that labeling the units of alcohol contained in a drink may carry unintended consequences, with the label being used as a reference cue to purchase stronger alcohol products (Jones & Gregory, 2009; Maynard et al., 2018). Furthermore, in the absence of verbal descriptors of alcohol strength drinkers tend to underestimate the alcohol units contained in their drinks, reflected in the pouring of larger servings (de Visser & Birch, 2012; Furtwängler & de Visser, 2016). Similar paradoxical effects have been found in relation to calorie labeling on alcohol containers, leading to increased intentions to have more alcohol (see Bui, Burton, Howlett, & Kozup, 2008) and using calorie information to reduce food consumption so that more alcohol might be drunk (Maynard et al., 2018). However, it remains unclear whether similar under- or overestimates can be observed when verbal descriptors of strength are shown.

To our knowledge only one study to date has examined consumers’ perceptions of strength (%ABV) and appeal of alcohol products using verbal descriptors of lower alcohol strength (Vasiljevic, Couturier, & Marteau, 2018). In a sample of 1,600 weekly wine and beer drinkers sampled from a nationally representative U.K. panel it was found that verbal descriptors of lower strength wine and beer form two clusters and effectively communicate reduced alcohol content. *Low*, *Lower*, *Light*, *Lighter*, and *Reduced* formed a cluster and were rated as denoting lower strength products than *Regular*, but higher strength than the cluster with intensifiers consisting of *Extra Low*, *Super Low*, *Extra Light*, and *Super Light*. Based on the number of significant pairwise comparisons between descriptors, both *Low* and *Super Low* were the most differentiated labels within the cluster of single adjectives and adjectives with intensifiers, respectively. In terms of appeal, *Regular* was rated the most appealing, with the low verbal descriptors using intensifiers rated least appealing.

Although this study was timely and informative with regard to how weekly drinkers perceive the communicated alcohol strength of different verbal descriptors of strength, and how appealing such labeled products are, the study was limited because the verbal descriptors were not coupled with %ABV indicators on actual labels. Consequently, the study neither speaks to consumers’ perceptions of alcohol products with varying %ABV, nor to how such perceptions may change when coupled with different verbal descriptors of strength. The present study aimed to fill this gap, by combining %ABV indicators with a selection of verbal descriptors identified as most differentiating and understandable in the study by Vasiljevic and colleagues (see Vasiljevic, Couturier, et al., 2018). Purposefully tailored labels were developed so that we could control for participants’ prior brand preferences.

Understanding the appeal of different labels combining verbal and numerical descriptors of strength is important for discerning the potential impact of such labels upon product selection and consumption. Appeal is an attitude, affective in origin, involving positive and negative feelings toward an object or behavior (Ajzen, 2001). Affect takes primacy in influencing much of our behavior (Strack & Deutsch, 2004). Pertinent to this study, prior research suggests that appeal of alcohol is predictive of alcohol consumption (see Morgenstern, Isensee, Sargent, & Hanewinkel, 2011). To ensure good understanding across the population (including those with low as well as high levels of education and numeracy), it is imperative that we also examine public understanding of the alcohol strength of products labeled with different verbal and numerical descriptors of alcohol content. If people underestimate calorie or unit content of drinks labeled as lower in alcohol strength, then they may overindulge in such products. Similarly, consumers’ perceptions as to whether a drink may be appropriate for consumption by children demonstrates the levels of perceived risks that people associate with alcohol labeled as lower in strength. All these indicators have important ramifications for any future legislative changes to the labeling rules of alcohol.

The primary aim of the present study was to assess the impact on product appeal of different verbal descriptors denoting lower strength, with and without %ABV, for (a) wine and (b) beer. Possible moderators of the relationship between verbal descriptors and %ABV on appeal were also examined, such as differences in sociodemographic indicators, numeracy levels, motivation to reduce consumption, and self-licensing. A secondary aim was to examine participants’ understanding of the labels in terms of communicated alcohol units, calorie content, the drink-driving limit, and appropriateness for the drink to be consumed by children.

## Method

### Participants

Three thousand, three hundred ninety adults (1,697 wine and 1,693 beer drinkers) completed the study. Participants were recruited by a market research agency. The initial sample that accessed the study was demographically similar in age, gender, SES, and geographical region as the U.K. population. Only those who reported drinking alcohol at least once per week were eligible to continue with the study. Furthermore, participants who failed attention checks were not permitted to complete the study. Allocation to the wine or beer arm was done according to drinking preference (see Procedure). For the characteristics of the sample see Table 1. The sample size provided 90% power at 5% level of significance to detect a medium-sized difference in product appeal between one “low alcohol” and another of the “low alcohol” verbal descriptors, taking into account multiple comparisons. These power calculations were based on differences in ratings of appeal observed in a pilot study (Vasiljevic, Couturier, & Marteau, 2015).[Table-anchor tbl1]

### Design

A 3 × 6 between-subjects experimental study (for wine and beer) in which participants were randomized to one of 18 groups with one of three levels of verbal descriptor (*Low* versus *Super Low* versus *No verbal descriptor*) and six levels of %ABV (five levels varying for wine and beer, and no level given).

### Labels

**Figure 1 fig1:**
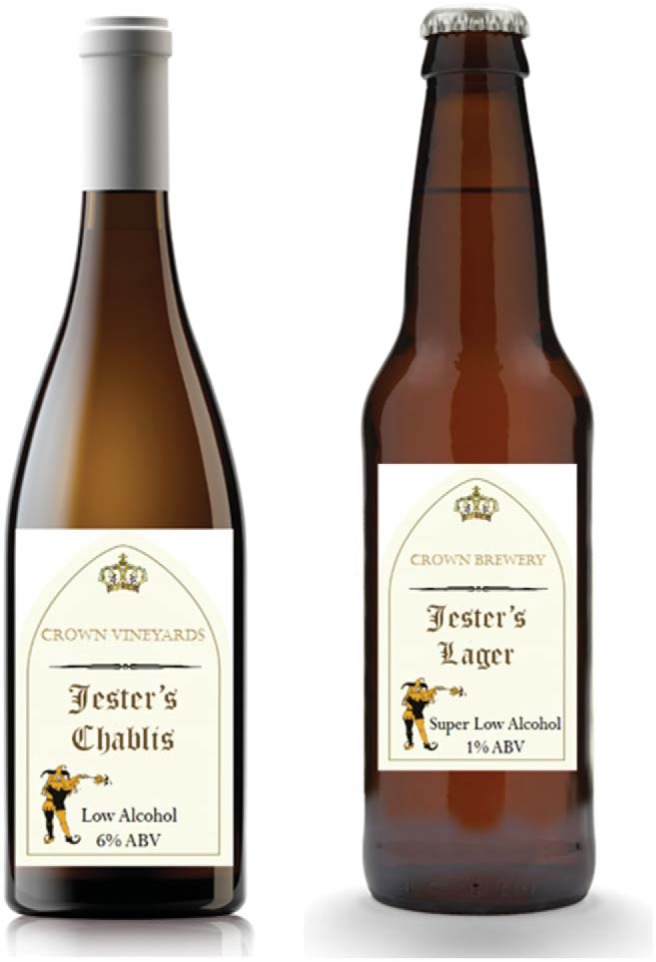
Sample of two lower strength alcohol labels seen by participants (one in wine, one in beer).

Two verbal descriptors of lower alcohol strength (*Low* and *Super Low*) were selected following a prior study on the basis of being reliably perceived as denoting products lower in alcohol strength when compared to the *Regular* verbal descriptor (Vasiljevic, Couturier, et al., 2018). In the present study, the impact of adding %ABV to these two verbal descriptors was assessed using a range of %ABV (wine: 0%, 4%, 6%, 8%, 10%; beer: 0%, 1%, 2%, 3%, 4%). As a control, we had labels with no %ABV given (see Figure 1 below for two sample labels, one for wine and one for beer). For the analyses, we considered that a label with *No verbal descriptor* and No %ABV corresponds to an average (Regular) strength product, that is, a standard drink available on the market - 12.9%ABV wine and 4.2%ABV beer (Department of Health, 2014).^1^ For the labels combining *Low* or *Super Low* verbal descriptors with No %ABV we used average perceptions of strength that were obtained in Vasiljevic, Couturier, et al. (2018) for wine (*Low* 6.7%, *Super Low* 3.5%) and beer (*Low* 2.7%, *Super Low* 1.3%).[Fig-anchor fig1]

### Measures

#### Primary outcome

##### Product appeal

Two items measured participants’ appeal of the product they saw: “How likely are you to buy this wine/beer?” and “How likely are you to drink this wine/beer?” (answered on scales ranging from 1 = *very unlikely* to 7 = *very likely*). Interitem reliability was good, *r*_*wine*_ = .92, *p* < .001; *r*_*beer*_ = .93, *p* < .001.

#### Secondary outcomes

##### Understanding of alcohol strength and calorie content

Four items measured participants’ understanding of the alcohol strength of the product. The first item gauged participants’ knowledge of whether the product they saw could be safely consumed by children: “This wine/beer can be safely drunk by children aged over 12. Do you agree with this statement?” Responses were recorded on a scale from 1 = *strongly disagree* to 7 = *strongly agree*. Participants’ responses were dichotomized whereby any level of disagreement with the statement was considered correct, and any level of agreement as incorrect.

The second item gauged participants’ understanding of how many drinks of the product they could have without exceeding the drink-driving legal limit: “How many small glasses (125 ml) of this wine/half-pints of this beer do you think you could have and still drive within the legal limit?” Responses were recorded on 0–20 slider scales. Slider scales are interactive versions of more traditional rating scales, allowing participants to give finer-grained responses (including decimal figures). To determine the accuracy of participants’ responses we calculated how many small glasses (125 ml) of wine or half-pints of beer participants could drink and still drive within the legal driving limit for U.K. (excluding Scotland). This was done for all levels of %ABV, compiling scores separately for men and women, and based on a person with average weight and metabolism (for more details on the calculations see online supplementary materials).

The third item gauged participants’ understanding of unit content of the drink they were shown: “How many units of alcohol do you think a small glass (125ml)/half-pint of this wine/beer would have?” Responses were recorded on 0–20 sliders. For analysis we determined the actual number of units contained in each of the drinks according to its %ABV (see online supplementary materials).

The fourth item assessed participants’ perceptions of how many drinks they could have of the given drink to match the alcohol contained in a drink of regular alcohol strength: “How many small glasses (125ml)/half-pints of this wine/beer do you think match the alcohol contained in a small glass (125ml)/half-pint of regular alcohol strength wine/beer?” Responses were recorded on 0–20 sliders. Taking into account the number of units as calculated for the item above, we determined how many small glasses/half-pints would match the alcohol contained in a small glass/half-pint of regular strength wine/beer (see online supplementary materials).

The fifth item gauged participants’ understanding of the calorie content of the drink they were randomized to see: “The recommended daily calorie intake from food and drinks for men is 2500 calories (kcal), and for women 2000 calories (kcal). How many calories (kcal) do you think a small glass (125ml)/half-pint of this wine/beer has?” Responses were open-ended, but constrained to responses ranging from 0–2500.

#### Individual difference measures

##### Risky drinking

This was assessed using the AUDIT-C (Bush, Kivlahan, McDonell, Fihn, & Bradley, 1998), the first three items of the Alcohol Use Disorders Identification Test (AUDIT; Babor, Higgins-Biddle, Saunders, & Monteiro, 2001). A sample item asked “How many drinks containing alcohol do you have on a typical day when you are drinking?” Responses ranged from *1 or 2*, *3 or 4*, *5 or 6*, *7 to 9*, *10 or more*. Following recommendations responses to the AUDIT-C were summed, and dichotomized to denote riskier (scoring above 5) versus less risky drinking patterns (scoring below 5; Public Health England, 2017).

##### Motivation to reduce consumption

This was assessed via three items (*“*Thinking about the next 6 months: I intend to drink less alcohol/I want to drink less alcohol/I will try to drink less alcohol”). Responses ranged from *1* (*Strongly disagree*) to *7* (*Strongly agree*; α_*wine*_ = .96; α_*beer*_ = .95).

##### Self-licensing

This was assessed with two items: “If I were to have a low alcohol drink, I would feel like I deserved to have something stronger for my next drink” and “If I were to have a low alcohol drink, I would feel like I could have more than my usual number of drinks.” The items were rated on 7-point scales from *1* (*Strongly disagree*) to *7* (*Strongly agree*; *r*_*wine*_ = .59, *p* < .001; *r*_*beer*_ = .49, *p* < .001).

##### Numeracy

This was assessed using a single item from Lipkus and colleagues’ (2001) Numeracy Scale (validated by Wright, Whitwell, Takeichi, Hankins, & Marteau, 2009): “Which of the following numbers represents the biggest risk of getting a disease: 1 in 100 risk of getting a disease, 1 in 1,000 risk of getting a disease, 1 in 10 risk of getting a disease?” For analyses, answers were dichotomized into those who answered correctly (high numeracy levels) versus those who answered incorrectly (low numeracy levels; for more details see Wright et al., 2009).

##### Demographic characteristics

The following were recorded: age, gender, ethnicity, and socioeconomic status (assessed using individual-level measures of highest educational qualification, income and occupational status, and neighborhood-level deprivation assessed from postcode information and transformed into an Index of Multiple Deprivation—IMD; see Oguz, Merad, & Snape, 2013).

### Procedure

The study received ethics approval from the University of Cambridge Psychology Research Ethics Committee (PRE.2015.077). Participants were recruited by a market research agency. Only those participants who reported drinking at least once a week were eligible to proceed with the study. Participants were randomized to see one of the 18 alcohol labels placed on a bottle of wine or beer after stating their alcohol preference. Participants who reported drinking wine and beer in equal proportions were randomly assigned to either the wine or beer surveys. Selection of the presented product with the label was randomly assigned by the survey software platform Qualtrics. With the product in view, participants completed the study outcome measures.

### Analysis

We performed a series of linear regressions for the primary outcome (product appeal). Secondary outcomes pertaining to understanding of strength and calorie estimation were analyzed using logistic regression. For the logistic regressions we combined the answers of those correctly estimating or underestimating/overestimating allowing us to test whether participants gave answers in line with a healthier/less risky outcome. Because the data showed some deviation from the normal distribution conditionally to the predictors, we obtained the parameter standard errors by means of a nonparametric bootstrap (Davison & Hinkley, 1997). For all analyses the intercept (comparison group) was the experimental condition denoting a Regular strength drink (labeled with *No verbal descriptor* and No %ABV). We also performed a series of sensitivity analyses. Similar results were obtained in these sensitivity analyses as with normal/regular inference because of the large sample size in both the wine and beer samples. For the regression analyses, we employed a multiplicity correction which takes into account the dependence between the regression parameters (Bretz, Hothorn, & Westfall, 2010). For all pairwise comparisons, we employed a Dunn-Šidák multiplicity correction because of the independence of the experimental groups. Both correction procedures enabled us to achieve a family-wise Type I error rate of 5% at the outcomes and drink level (global).

## Results

### Primary Outcome

#### Product appeal

**Figure 2 fig2:**
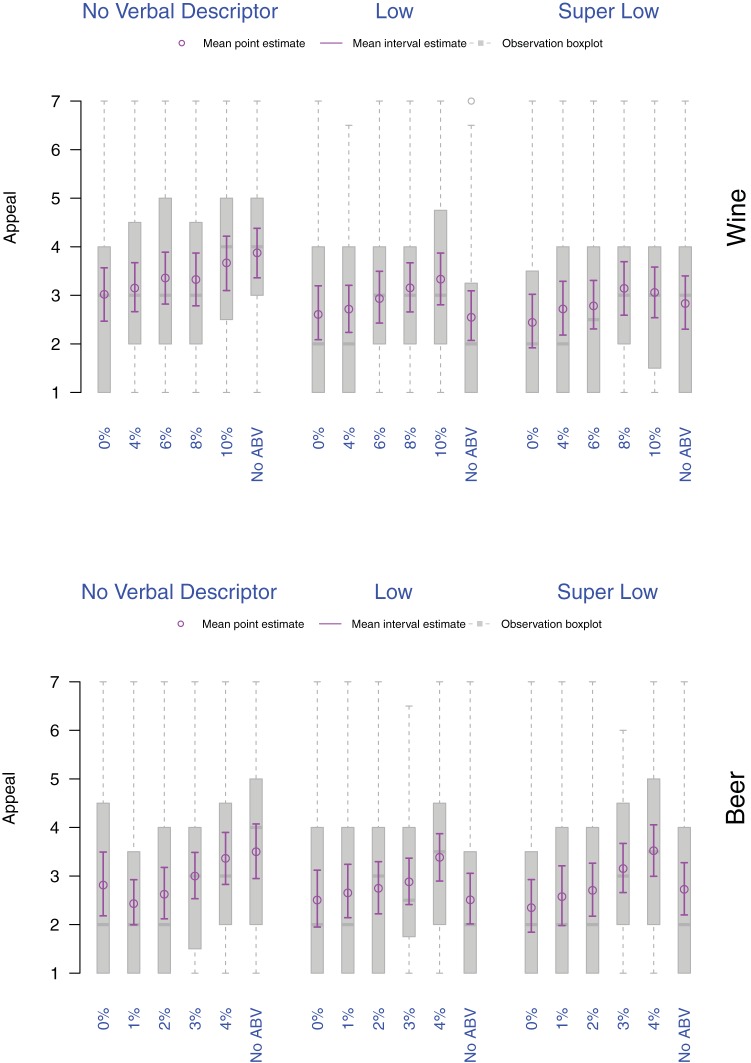
Appeal as a function of drink (top: Wine, bottom: Beer), verbal descriptor, and %ABV. The boxplots of participants’ scores appear in gray. Violet dots and arrows, respectively correspond to the point and interval estimates for the mean appeal.

Appeal increased with %ABV and was highest for products without a verbal descriptor and without %ABV (corresponding to a Regular [average] strength wine or beer product that would be expected to be on the market; see Figure 2). Appeal decreased significantly as %ABV decreased with lowest appeal for wine with 0%ABV and 4%ABV, and for beer with 1%ABV and 2%ABV (*p*s ≤ .001, for the comparison with Regular). For *Low* verbal descriptors, appeal was lowest when combined with No %ABV, and for *Super Low* verbal descriptors appeal was lowest when combined with 0%ABV. Both *Low* and *Super Low* verbal descriptors had a similar detrimental impact on appeal (*p*s_wine_ < .001; *p*s_beer_ < .002). Adding %ABV to alcohol drinks labeled with verbal descriptors denoting lower strength products (*Low* or *Super Low*) increased their appeal slightly although this remained low. When controlling for multiple comparisons this increase in appeal was only statistically significant for labels combining the verbal descriptor *Low* with higher %ABVs (wine: 8%ABV and 10% ABV, *p*s ≤ .003; beer: 1%ABV, 3%ABV, and 4%ABV, *p*s ≤ .002). At the same time, pairwise comparisons indicated that none of the combinations involving %ABV and verbal descriptors *Low* and *Super Low* stood out in terms of having a larger impact on appeal than other combinations (for the regressions see online supplementary materials).[Fig-anchor fig2]

#### Moderators of product appeal

A series of linear regression analyses probing for three-way interactions between verbal descriptor and %ABV and the demographic/individual difference measures (age, gender, ethnicity, SES, risky drinking, motivation to reduce consumption, self-licensing, and numeracy) demonstrated that the effects of the independent variables were not moderated by any of the demographic or individual difference measures.

### Secondary Outcomes

#### Understanding of alcohol strength

##### Item 1 – Level of perceived appropriateness for children to consume given drink

**Figure 3 fig3:**
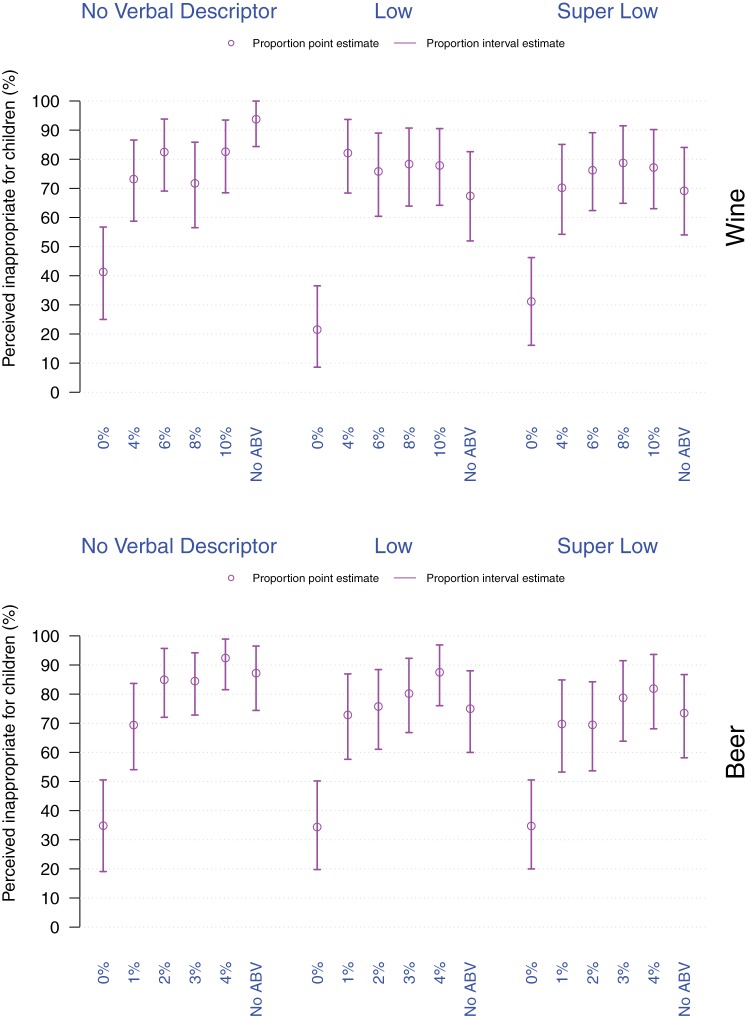
Proportion of participants perceiving a drink inappropriate for consumption by children as a function of drink (top: Wine, bottom: Beer), verbal descriptor, and %ABV. Violet dots and arrows, respectively correspond to the point and interval estimates for the proportion of participants perceiving a given drink as inappropriate for children.

As can be seen in Figure 3, the majority of participants judged the Regular strength wine (94%) and beer (87%) as inappropriate for children. For wine, the highest proportion perceiving the drink inappropriate for children was given when participants saw the wine with *No verbal descriptor* and No %ABV, that is, a standard bottle of wine (Regular). For beer, the highest proportion perceiving the drink inappropriate for children was given when participants saw the beer with *No verbal descriptor* and 4%ABV, followed by a beer with *No verbal descriptor* and No %ABV and a beer with a *Low* verbal descriptor with 4%ABV label.[Fig-anchor fig3]

Although the majority of participants judged that any alcohol-containing wine and beer was inappropriate for children to consume, the inclusion of %ABV or verbal descriptors denoting lower alcohol strength decreased the proportion of people who judged the drinks to be inappropriate for consumption by children when compared with the Regular strength drink. This pattern emerged mainly for wine (0%ABV, 4%ABV, and 8%ABV, *p*s < .001), but not for beer. The majority of participants (>65%) indicated that drinks with 0%ABV are appropriate for children.

##### Item 2 – Knowledge of drinks suitable for driving within the legal limit

**Figure 4 fig4:**
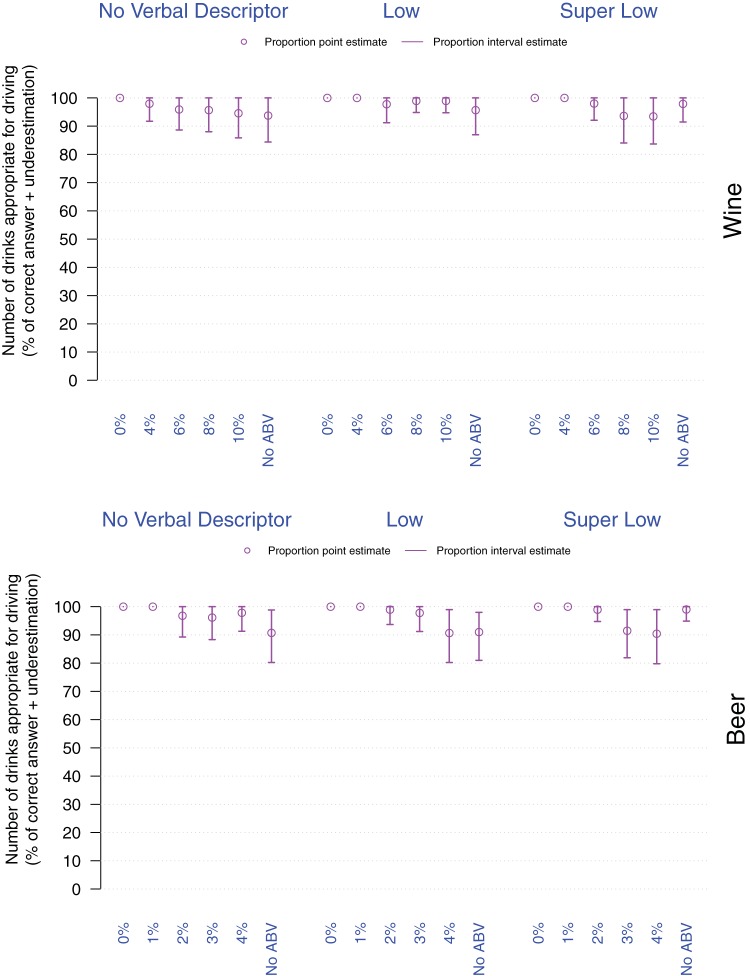
Proportion of participants correctly estimating or underestimating the number of drinks suitable for driving within the legal limit as a function of drink (top: Wine, bottom: Beer), verbal descriptor, and %ABV. Violet dots and arrows, respectively correspond to the point and interval estimates for the proportion of participants correctly estimating or underestimating the appropriate number of drinks for driving within the legal limit.

People mostly underestimated the number of drinks suitable for driving within the legal limit (see Figure 4). Although the regressions revealed no reliable differences compared with a Regular (average) strength drink, descriptively this underestimation appeared to increase as %ABV decreased, which is in part an artifact of the methodology as the lowest possible value increases with increasing %ABV. However, it is important to note that the variance seemed to decrease when the %ABV increased, suggesting that the uncertainty about the suitable number of drinks for driving within the legal limit decreased for %ABV values that are closer to standard (Regular) wines/beers. Whereas the statistical model shows that all wine drinkers consistently underestimate the number of drinks suitable for driving within the legal limit, the model also indicates that a small minority of beer drinkers (approximately 9%) overestimate the number of drinks suitable for driving, but only for the following beer products: *No verbal descriptor* and No %ABV, *Low* verbal descriptor and No %ABV *or* 4%ABV, and *Super Low* verbal descriptor and 3%ABV *or* 4% ABV.[Fig-anchor fig4]

##### Item 3 – Understanding of units contained in a small glass (125 ml)/half-pint of a given drink

**Figure 5 fig5:**
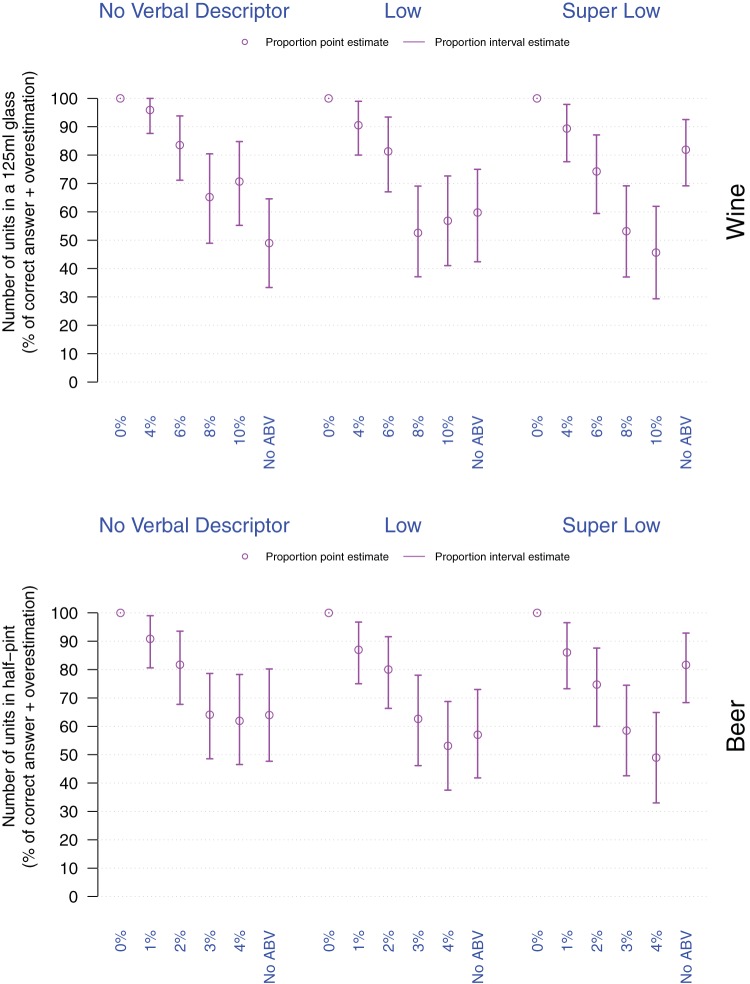
Proportion of participants correctly estimating or overestimating the number of units of alcohol contained in a small glass (125 ml)/half-pint as a function of drink (top: Wine, bottom: Beer), verbal descriptor, and %ABV. Violet dots and arrows, respectively correspond to the point and interval estimates for the proportion of correct estimates and overestimates.

Participants were either correct or overestimated the number of units of alcohol contained in a small glass (125 ml)/half-pint of the shown wine/beer provided that the label referred to 0%ABV, or to a percentage ABV closer to 0%ABV (4%ABV for wine, 1% and 2%ABV for beer, and for both wine and beer in the case of *Super Low* also when combined with No %ABV]) Thus, drinks labeled with lower %ABV yielded a higher proportion of correct answers or overestimates (see Figure 5 below and online supplementary materials).[Fig-anchor fig5]

Logistic regressions revealed that overall in wine labels with *Super Low* verbal descriptors, and labels with 4% or 6%ABV (*p*s < .001), and in beer labels with 1% or 2%ABV (*p*s ≤ .008) yielded different unit estimates compared with the Regular strength drink.

##### Item 4 – Understanding of the number of small glasses (125 ml)/half-pints of a given drink that would match the alcohol contained in a small glass (125 ml)/half-pint of regular alcohol strength wine/beer

**Figure 6 fig6:**
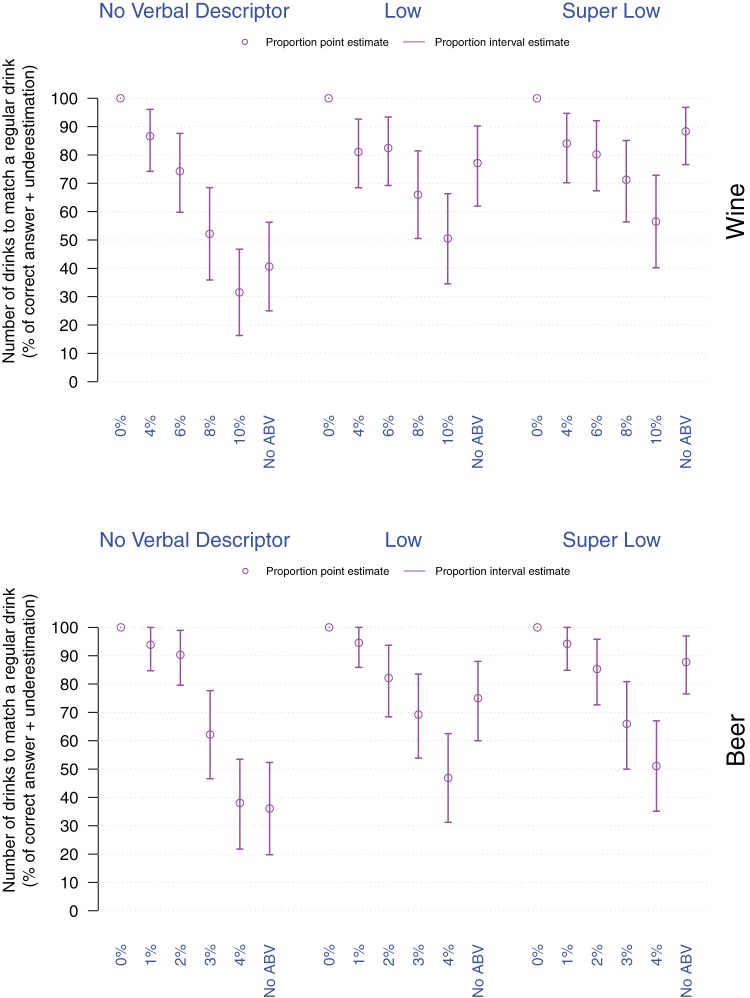
Proportion of participants correctly estimating or underestimating the number of small glasses/half-pints that would match the alcohol contained in a small glass (125 ml)/half-pint of regular alcohol strength drink as a function of drink (top: Wine, bottom: Beer), verbal descriptor, and %ABV. Violet dots and arrows, respectively correspond to the point and interval estimates for the proportion of correct estimates and underestimates.

Participants were either correct or underestimated the number of small glasses/half-pints that would match the alcohol contained in a small glass (125 ml)/half-pint of regular alcohol strength wine/beer with the exception of wine labeled with *No verbal descriptor* combined with 10%ABV which yielded overestimates. Logistic regressions revealed that overall in wine labels with *Low* or *Super Low* verbal descriptors, and labels with 4% or 6%ABV (*p*s < .001), and in beer labels with *Low* or *Super Low* verbal descriptors and labels with 1%, 2% or 3%ABV (*p*s < .001) yielded underestimates of the number of small glasses/half-pints needed to match the alcohol contained in a small glass/half pint of regular alcohol strength wine/beer compared to the Regular strength drink [see Figure 6].[Fig-anchor fig6]

#### Calorie estimates

**Figure 7 fig7:**
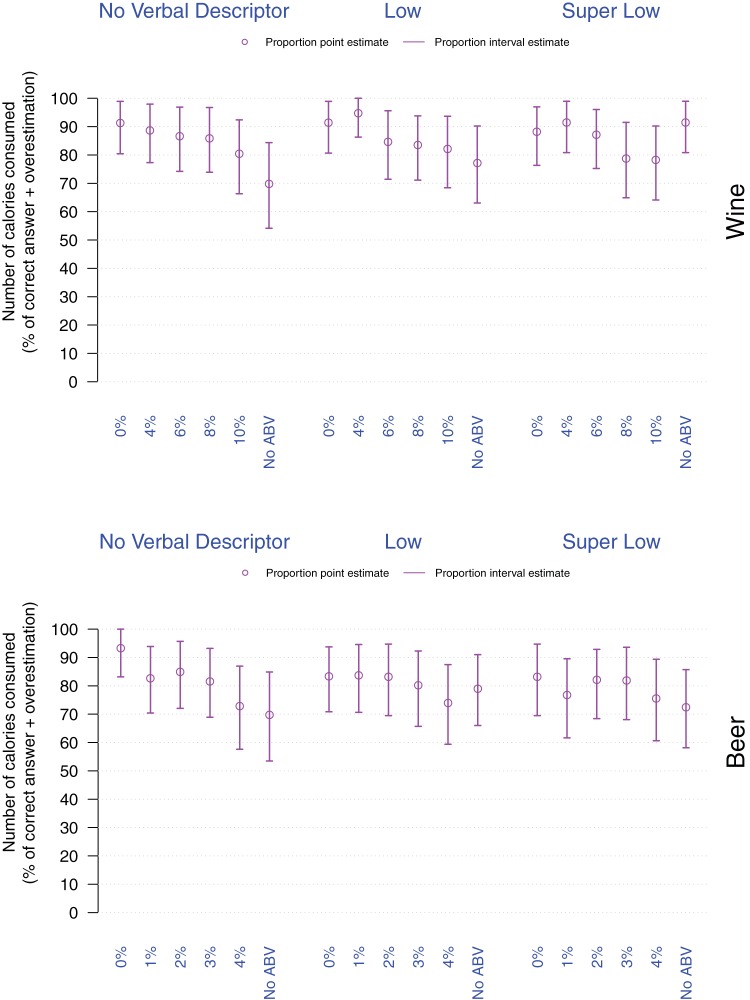
Proportion of participants correctly estimating or overestimating the calorie content of a given drink as a function of drink (top: Wine, bottom: Beer), verbal descriptor, and %ABV. Violet dots and arrows, respectively correspond to the point and interval estimates for the proportion of correct estimates and overestimates.

First, we compared the actual and estimated number of calories contained in the drinks presented to participants, which revealed that participants overestimated the calorie content to a similar extent across all drinks. Considering the large spread of responses, we also examined the proportion of participants who made accurate estimates or overestimated the calorie content (vs. those who made underestimates). We then submitted these proportions to logistic regressions indicating that relative to the Regular strength drinks, more people overestimated the calorie contents of wine labeled with *Super Low* verbal descriptor, or with 0% or 4%ABV; and of beer labeled with 0%ABV; *p*s < .001 (see Figure 7 and online supplementary materials).[Fig-anchor fig7]

## Discussion

The U.K. national regulations covering the use of lower strength alcohol terms were repealed at the end of December 2014, with a sunset clause in place until the end of 2018. Proposed legislative changes include extending the range of verbal descriptors denoting low/er alcohol strength, and increasing the strength limit to include products lower than the current average on the market but higher than the current legislated cap of 1.2%ABV. The current study assessed the impact on product appeal and understanding of strength of labeling wine and beer with different verbal descriptors denoting lower strength, with and without %ABV.

Products labeled with verbal descriptors denoting lower alcohol content (*Low* and *Super Low*) had lower appeal than Regular (average) strength products. Appeal decreased as %ABV decreased, with lowest appeal found for wine with 0%ABV and 4%ABV, and for beer with 1%ABV and 2%ABV. Adding %ABV to verbal descriptors denoting lower strength alcohol products (*Low* and *Super Low*) increased their low appeal to some extent, in particular for labels combining the verbal descriptor *Low* with %ABV larger than zero. Participants’ demographic and individual difference characteristics did not modify these effects.

These results mirror current sales data by showing that consumers prefer regular (average) strength wines and beers (Department of Health, 2014). The study extends recent findings by Vasiljevic, Couturier, et al. (2018), by showing that the appeal of labels denoting alcohol strength decreases with decreasing communicated strength, regardless of whether strength is communicated verbally and/or numerically (%ABV). The results are compatible with the possibility that lower strength alcohol labeling could be used akin to alcohol unit labeling, with consumers using the labels to choose stronger products (Jones & Gregory, 2009; Maynard et al., 2018).

Participants’ age, gender, ethnicity, SES, risky drinking habits, motivation to reduce consumption, self-licensing, and numeracy levels did not moderate the effects of lower strength verbal descriptors and %ABV on product appeal. This pattern of results suggests that lower strength alcohol labeling (both verbal and numerical) may have similar effects across different demographic groups in the population. This is encouraging in terms of any changes to the alcohol labeling regulations regarding lower alcohol content.

Understanding of strength and calorie content was generally high across the various drinks with the majority of participants correctly identifying or erring on the side of caution regarding consumption of the products by children above 12, drinking within the legal driving limit, the number of units in a given drink, the amount of calories in a drink, and the number of glasses required to match a *Regular* strength drink. These findings suggest that self-reported understanding of the strength and calorie content of drinks denoting different alcohol strengths is good, with no detrimental impact arising from lower strength labeling. The results hint at the possibility that verbal and numerical descriptors of alcohol strength may be easier for consumers to understand, unlike alcohol unit labeling and calorie labeling which are often poorly understood by consumers (see Bui et al., 2008; de Visser & Birch, 2012; Furtwängler & de Visser, 2016; Maynard et al., 2018). However, because our study did not measure actual consumption, there are uncertainties as to whether this self-reported understanding of strength and calorie content would result in nonharmful consumption. Further studies measuring behavioral outcomes, including consumption are warranted.

### Strengths and Limitations With Future Directions

This is the first study to examine the impact of labeling wines and beers with verbal and numerical information of lower alcohol strength on product appeal and understanding of strength. The use of questions with factual answers to gauge participants’ understanding of alcohol strength further enhances the validity of the present findings.

The study is further strengthened by using a large sample of weekly wine and beer drinkers sampled from the general population of the U.K. Given the popularity of lower strength alcohols in other high income countries (“Big brewers see strong potential for weak beer,” 2016; Wine Intelligence, 2013), replications with samples drawn from non-U.K. contexts are needed to further elucidate the effects found in this study. The findings may also be applicable to cultural contexts where abstinence levels are high (Africa and Gulf countries), where lower strength products may be positioned as entry level products to expand the market and reduce abstinence (Babor et al., 2010). Replications will be necessary to gauge the impact of lower strength alcohol labeling on population health across different cultural contexts.

The study is limited by assessing participants’ perceptions and not behavioral responses to products with lower strength alcohol labels. Although such perceptions may predict behavioral responses (Ajzen, 2001), the strength of this prediction is unknown in the current context. Future studies should extend the current findings using behavioral outcomes, including purchasing and consumption.

Importantly, some existing alcohol labeling has used similar verbal terms to the ones used in this study (e.g., *low*) to refer to reduced calorie content, rather than reduced alcohol content. In our study we explicitly told participants that the labels referred to alcohol strength. Policymakers should bear this in mind and avoid possible misunderstandings by including terms such as Alcohol (e.g., “*Low Alcohol*”) or Strength (e.g., “*Low Strength*”) to explicitly inform consumers that the labeling refers to alcohol strength.

Although we were able to control for prior brand preferences by custom-making novel fictitious labels, future research should extend these findings and examine the impact of lower strength alcohol labeling in conjunction with existing branding. Whether the effects of lower strength alcohol labeling would be enhanced or diminished when coupled with branding is currently unknown.

### Policy Implications

With the view of aiding decision-making in the context of imminent legislative changes to alcohol labeling rules in the U.K., the present study aimed to examine the impact of labels denoting lower alcohol content on appeal and understanding of strength among weekly wine and beer drinkers (Department of Health, 2012). Proposed legislative changes include extending the variety of verbal descriptors that could be used to denote lower alcohol content, and extending the strength limit to include products lower than the current average on the market but higher than the current legislated cap of 1.2%ABV.

It is difficult to infer any impact of lower strength alcohol labeling on consumption. Such labeling reduced the appeal of products which suggests that any changes to the legislative framework regarding lower content labeling may not result in overall reductions of alcohol consumption at the population level. Policy options other than explicit labeling of lower strength alcohols may be more effective at encouraging consumers to switch to lower content alternatives. These include preferential tax treatment for lower content alcohols, resulting in reduced price per container while at the same time not highlighting the lower alcohol content of the products.

Further assessment of the impact of lower strength alcohol labeling is clearly warranted. Such assessments should evaluate not only how people respond to these labels (including consumption) but also, by taking a whole systems approach, how the alcohol industry and retailers respond through branding and marketing (see Petticrew et al., 2017; Vasiljevic, Coulter, et al., 2018).

## Supplementary Material

10.1037/adb0000376.supp

## Figures and Tables

**Table 1 tbl1:** Participant Demographic and Other Characteristics

Characteristic	Drink	
	Wine (*n* = 1,697)	Beer (*n* = 1,693)
Gender		
Male	611 (36)	1262 (75)
Female	1086 (64)	431 (25)
Age group		
18–35	207 (12)	253 (15)
35–45	295 (18)	308 (18)
45–60	560 (33)	641 (38)
60–99	635 (37)	491 (29)
Education^a^		
4 GCSEs	255 (15)	341 (20)
1 A-level	310 (18)	285 (17)
2+ A-levels	287 (17)	305 (18)
University	781 (46)	688 (41)
N/A	64 (4)	74 (4)
Income^b^		
0–15.5K	306 (18)	358 (21)
15.5–25.5K	290 (17)	301 (18)
25–40K	499 (30)	446 (26)
>40K	497 (29)	500 (30)
N/A	105 (6)	88 (5)
Social grade		
Low	167 (10)	165 (10)
Medium	328 (19)	303 (18)
High	203 (12)	172 (10)
N/A	999 (59)	1053 (62)
Index of Multiple Deprivation (IMD)^c^		
Quintile 1	230 (14)	284 (17)
Quintile 2	263 (15)	280 (16)
Quintile 3	307 (18)	267 (16)
Quintile 4	268 (16)	250 (15)
Quintile 5	271 (16)	267 (16)
N/A	358 (21)	345 (20)
Riskier drinkers		
No	997 (58.8)	750 (44)
Yes	695 (41)	942 (55.9)
N/A	5 (0.2)	1 (0.1)
Numeracy^d^		
Correct	1217 (72)	1239 (73)
Incorrect	480 (28)	454 (27)
Ethnicity		
White	1592 (94)	1580 (93.5)
Other	97 (5.6)	104 (6)
N/A	8 (0.4)	9 (0.5)
*Note*. Percentages appear in parentheses.		
^a^ GCSEs (General Certificate of Secondary Education) are usually taken at age 15–16 in the UK; A-Levels at age 17–18. ^b^ Income bands are expressed per annum. ^c^ Index of Multiple Deprivation (IMD) denotes neighborhood-level deprivation; Quintile 1 reflects the highest level of deprivation and Quintile 5 the lowest level of deprivation. ^d^ Numeracy was measured with a single item taken from Lipkus, Samsa, and Rimer (2001) Numeracy Scale assessing participants’ understanding of the risk of getting a disease; answers were dichotomized into those who correctly versus those who incorrectly answered the question.		

## Footnotes

1 The recent study by Vasiljevic, Couturier, and Marteau (2018) reported that weekly wine and beer drinkers were able to correctly gauge the %ABV of wines and beers denoted as regular strength. Hence, we reasoned that if participants are presented with a product labeled without verbal or numerical information on strength, they will assume that the product denotes a regular (average, standard) strength wine/beer available on the market.
