# Supplementary material for: Impact on Product Appeal of Labeling Wine and Beer With (a) Lower Strength Alcohol Verbal Descriptors and (b) Percent Alcohol by Volume (%ABV): An Experimental Study
Source: Psychol Addict Behav. 2018 Aug 30;32(7):779–91. doi: 10.1037/adb0000376 (PMC6241458; doi:10.1037/adb0000376)
Supplement: Supplementary file 1 [file LowAlcS2OnlineSupplementsDeanonymisedForPublication26July2018.zip › LowAlcS2OnlineSupplementsDeanonymisedForPublication26July2018.pdf]

ONLINE SUPPLEMENTARY MATERIALS:

Impact on Product Appeal of Labeling Wine and Beer With (a) Lower Strength Alcohol Verbal Descriptors and (b) Percent Alcohol by Volume (%ABV): An Experimental Study

Milica Vasiljevic, Dominique-Laurent Couturier, & Theresa M. Marteau

Behaviour and Health Research Unit, University of Cambridge, UK

Supplement I: Transformations/calculations of variables....................................pp. 2-7

Supplement II: Linear/logistic regression estimates with descriptives...............pp. 8-25

Supplement I:
Transformations/calculations of variables

Item 2 – level of understanding as assessed for knowledge of drinks suitable for driving within the legal limit:
The question was worded as: **How many small glasses (125 ml) of this wine/half-pints of this beer do you think you could have and still drive within the legal limit? [0-20 scale on a slider]**

We first ascertained the current drink-driving limit in the UK excluding Scotland (https://www.gov.uk/drink-drive-limit):

**Level of alcohol**	England, Wales and Northern Ireland
Micrograms per 100 millilitres of breath	35
Milligrammes per 100 millilitres of blood	80
Milligrammes per 100 millilitres of urine	107

We then calculated the blood alcohol content for an average male and female if they consumed a small glass (125ml)/half-pint of a wine/beer with a given %ABV. For the different BAC calculations in wine and beer see Table S1a and S1b below.

Table S1a & b: BAC for an average male and female after consuming a small glass of wine/half-pint of beer.

Wine (% ABV)	Male	Female
0%	0	0
4%	0.007	0.009
6%	0.01	0.014
8%	0.013	0.019
10%	0.016	0.023
No% No Verbal Label [12.9%]	0.021	0.03
No% Low Alc [6.7%]	0.011	0.015
No% Super Low Alc [3.5%]	0.006	0.008

Beer (% ABV)	Male	Female
0%	0	0
1%	0.004	0.005
2%	0.007	0.011
3%	0.011	0.016
4%	0.015	0.021
No% No Verbal Label [4.2%]	0.016	0.022
No% Low Alc [2.7%]	0.01	0.014
No% Super Low Alc [1.3%]	0.005	0.007

Taking into account the legal drink-driving limit in the UK (excl. Scotland) we then worked out the correct answer of how many small glasses (125ml)/half-pints of wine/beer with a given %ABV one could consume and still drive with the legal limit. These figures are shown in Table S2a & S2b below. For the logistic regression we combined the proportion of participants correctly estimating or underestimating *vs.* those overestimating the number of drinks one could have and still drive within the legal limit. Combining the answers of those who correctly estimated and underestimated allowed us to test whether participants gave answers in line with a healthier/less risky outcome.

Table S2a & b: Limit of small glasses of wine/half-pints of beer one can drink and still drive within the legal limit.

Wine (% ABV)	Male	Female
0%	20	20
4%	11	8
6%	8	5
8%	6	4
10%	5	3
No% No Verbal Label [12.9%]	3	2
No% Low Alc [6.7%]	7	5
No% Super Low Alc [3.5%]	13	10

Beer (% ABV)	Male	Female
0%	20	20
1%	20	16
2%	11	7
3%	7	5
4%	5	3
No% No Verbal Label [4.2%]	5	3
No% Low Alc [2.7%]	8	5
No% Super Low Alc [1.3%]	16	11

Note. For 0% ABV infinity is possible, however our answer scale only went from 0-20, hence 20 is considered as the correct answer.

Item 3 – understanding of units contained in a small glass (125ml)/half pint of a given drink:

The question was worded as: **"How many units of alcohol do you think a small glass (125ml)/half-pint of this wine/beer would have?" [0-20 slider scale]**

We first calculated the actual units contained in each of the drinks according to its %ABV. The formula used for these calculations was strength (ABV) x volume (ml) ÷ 1,000 = units. The below tables show how many units are contained in a small glass (125ml) of wine or half-pint of beer with the different %ABV used in the study design. We then used these figures to calculate whether participant's responses were correct, underestimation or overestimation of the correct figure (see Table 3a & b below). For the logistic regression we combined the proportion of participants correctly estimating or overestimating *vs.* those underestimating the number of units in a small glass/half-pint. Combining the answers of those who correctly estimated and overestimated for this question allowed us to test whether participants gave answers in line with a healthier/less risky outcome.

Table S3a & b: Units of alcohol contained in a small glass of wine/half-pint of beer.

Wine (% ABV)	Units in 125ml
0%	0
4%	0.5
6%	0.8
8%	1
10%	1.3
No% No Verbal Label [12.9%]	1.6
No% Low Alc [6.7%]	0.8
No% Super Low Alc [3.5%]	0.4

Beer (% ABV)	Units in 1/2 pint
0%	0
1%	0.3
2%	0.6
3%	0.9
4%	1.1
No% No Verbal Label [4.2%]	1.2
No% Low Alc [2.7%]	0.8
No% Super Low Alc [1.3%]	0.4

Item 4 – understating of the number of small glasses (125 ml)/half-pints of a given drink would match the alcohol contained in a small glass (125 ml)/half-pint of regular alcohol strength wine/beer:

The question was worded as: **"How many small glasses (125 ml)/half-pints of this wine/beer do you think match the alcohol contained in a small glass (125 ml)/half-pint of regular alcohol strength wine/beer?" [0-20 slider scales]**

Considering the correct number of units contained in a small glass (125ml) of wine or half-pint of beer according to the %ABV given in the study design (see the Tables S4a & S4b below), we used those values to calculate how many small glasses/half-pints would match the alcohol contained in a small glass/half-pint of regular strength wine/beer. The below tables show how many small glasses/half-pints match a regular strength small glass/half-pint for the different %ABV used in our study design. For the logistic regression we combined the proportion of participants correctly estimating or underestimating *vs.* those overestimating the number of small glasses/half-pints to match a regular strength small glass/half-pint. Combining the answers of those who correctly estimated and underestimated for this question allowed us to test whether participants gave answers in line with a healthier/less risky outcome.

Table S4a & b: Number of small glasses/half-pints matching a regular strength small glass of wine/half-pint of beer.

Wine (% ABV)	No. of 125ml glasses
0%	20 [max. scale point]
4%	3.2
6%	2.2
8%	1.6
10%	1.3
No% No Verbal Label [12.9%]	1
No% Low Alc [6.7%]	1.9
No% Super Low Alc [3.5%]	3.7

Beer (% ABV)	No. of 1/2 pints
0%	20 [max. scale point]
1%	4.25
2%	2.1
3%	1.4
4%	1
No% No Verbal Label [4.2%]	1
No% Low Alc [2.7%]	1.5
No% Super Low Alc [1.3%]	3.2

Calorie content:

Assessed by one item: **"The recommended daily calorie intake from food and drinks for men is 2500 Calories (kcal), and for women 2000 Calories (kcal). How many Calories (kcal) do you think a half-pint of this beer has?" [Responses were open-ended, but constrained to responses ranging from 0-2500]**

We worked out the correct answer presented in the table below for each of the %ABV conditions. We used the following formula for these calculations:

$$\frac{\text{volume (ml) x alcohol (ABV \%) x 8}}{1000}$$

Multiplying this answer by seven gives the approximate calorie content.
Calories from Carbohydrates (sugar): 4 calories per gram.

Read more:
http://www.decanter.com/learn/wine-terminology/how-to-count-the-calories-in-wine-ask-decanter-296021/#5R04bqcO4ze88Hfz.99

Table S5a & b: Calories in a small glass of wine/half-pint of beer.

Medium White Wine									
Volume (ml)	100	125	125	125	125	125	125	125	125
%ABV	12.9	12.9	10	8	6	4	6.7	3.5	0
Calories from %ABV	72.24	90.3	70	56	42	28	46.9	24.5	0
Calories from Sugar	12	15	15	15	15	15	15	15	15
Calories Total	84.24	105.3	85	71	57	43	61.9	39.5	15

Beer, Regular									
Volume (ml)	100	284.131	284.131	284.131	284.131	284.131	284.131	284.131	284.131
%ABV	4.2	4.2	4	3	2	1	2.7	1.3	0
Calories from %ABV	23.52	66.82761	63.64534	47.73401	31.82267	15.91134	42.96061	20.68474	0
Calories from Sugar	14.156	40.22158	40.22158	40.22158	40.22158	40.22158	40.22158	40.22158	40.22158
Calories Total	37.676	107.0492	103.8669	87.95559	72.04426	56.13292	83.18219	60.90632	40.22158

For the logistic regression we combined the proportion of participants correctly estimating or overestimating *vs.* those underestimating the number of calories in a small glass/half-pint. Combining the answers of those who correctly estimated and overestimated for this question allowed us to test whether participants gave answers in line with a healthier/less risky outcome.

Supplement II:
Linear/logistic regression estimates with descriptives

Product appeal

Table S6a. *Linear regression model on product appeal in wine drinkers.*

Experimental Condition	Estimate	Std. Error [boot]	z value [boot]	Pr(>|z|) [boot]	Sig	Sig (global)
(Intercept)	3.875	0.157	24.677	<0.0001	***	TRUE
Low label	-1.326	0.229	-5.795	<0.0001	***	TRUE
Super Low label	-1.045	0.235	-4.446	<0.0001	***	TRUE
ABV 0%	-0.853	0.237	-3.596	0.0003	***	TRUE
ABV 4%	-0.726	0.224	-3.242	0.0012	**	TRUE
ABV 6%	-0.519	0.231	-2.250	0.0244	*	FALSE
ABV 8%	-0.549	0.235	-2.339	0.0193	*	FALSE
ABV 10%	-0.207	0.234	-0.882	0.378		FALSE
Low:0%	0.912	0.337	2.705	0.0068	**	FALSE
SuperLow:0%	0.464	0.343	1.353	0.1761		FALSE
Low:4%	0.892	0.320	2.787	0.0053	**	FALSE
SuperLow:4%	0.614	0.335	1.830	0.0673	.	FALSE
Low:6%	0.904	0.333	2.720	0.0065	**	FALSE
SuperLow:6%	0.472	0.329	1.432	0.1521		FALSE
Low:8%	1.155	0.327	3.531	0.0004	***	TRUE
SuperLow:8%	0.863	0.336	2.564	0.0103	*	FALSE
Low:10%	0.989	0.334	2.962	0.0031	**	TRUE
SuperLow:10%	0.437	0.335	1.303	0.1927		FALSE

Note. Global significance level ($p < .05$) when correcting for multiple comparisons. Only effect estimates denoted with TRUE in the column Sig (global) are significant when correcting for multiple comparisons. The intercept (comparison group) is the experimental condition denoting a regular strength drink (labelled with No verbal descriptor and No %ABV).

Table S6b. *Linear regression model on product appeal in beer drinkers.*

Experimental Condition	Estimate	Std. Error [boot]	z value [boot]	Pr(>|z|) [boot]	Sig	Sig (global)
(Intercept)	3.500	0.178	19.673	<0.0001	***	TRUE
Low label	-0.990	0.241	-4.101	<0.0001	***	TRUE
Super Low label	-0.776	0.246	-3.151	0.0016	**	TRUE
ABV 0%	-0.685	0.275	-2.496	0.0126	*	FALSE
ABV 1%	-1.066	0.231	-4.619	<0.0001	***	TRUE
ABV 2%	-0.876	0.244	-3.593	0.0003	***	TRUE
ABV 3%	-0.500	0.238	-2.099	0.0358	*	FALSE
ABV 4%	-0.136	0.246	-0.551	0.5815		FALSE
Low:0%	0.681	0.371	1.833	0.0668	.	FALSE
SuperLow:0%	0.308	0.364	0.848	0.3967		FALSE
Low:1%	1.209	0.332	3.644	0.0003	***	TRUE
SuperLow:1%	0.917	0.346	2.654	0.008	**	FALSE
Low:2%	1.114	0.334	3.334	0.0009	***	TRUE
SuperLow:2%	0.857	0.343	2.497	0.0125	*	FALSE
Low:3%	0.869	0.325	2.670	0.0076	**	FALSE
SuperLow:3%	0.930	0.334	2.781	0.0054	**	FALSE
Low:4%	1.011	0.331	3.053	0.0023	**	TRUE
SuperLow:4%	0.933	0.339	2.750	0.006	**	FALSE

Note. Global significance level ($p < .05$) when correcting for multiple comparisons. Only effect estimates denoted with TRUE in the column Sig (global) are significant when correcting for multiple comparisons. The intercept (comparison group) is the experimental condition denoting a regular strength drink (labelled with No verbal descriptor and No %ABV).

Table S7. *Mean (95% CI) estimates across the 18 experimental conditions on product appeal in wine and beer drinkers*.

		Wine		Beer	
Verbal Descriptor	ABV [Wine; Beer]	*M*	95% CI	*M*	95% CI
No Verbal Descriptor	0%; 0%	3.022	(2.467, 3.566)	2.815	(2.180, 3.494)
No Verbal Descriptor	4%; 1%	3.149	(2.664, 3.671)	2.434	(1.995, 2.923)
No Verbal Descriptor	6%; 2%	3.356	(2.820, 3.889)	2.624	(2.118, 3.177)
No Verbal Descriptor	8%; 3%	3.326	(2.783, 3.871)	3.000	(2.532, 3.485)
No Verbal Descriptor	10%; 4%	3.668	(3.098, 4.217)	3.364	(2.826, 3.898)
No Verbal Descriptor	No ABV; No ABV	3.875	(3.359, 4.380)	3.500	(2.948, 4.071)
Low	0%; 0%	2.608	(2.085, 3.195)	2.505	(1.948, 3.121)
Low	4%; 1%	2.716	(2.237, 3.205)	2.652	(2.141, 3.240)
Low	6%; 2%	2.934	(2.429, 3.496)	2.747	(2.221, 3.296)
Low	8%; 3%	3.155	(2.658, 3.670)	2.879	(2.412, 3.368)
Low	10%; 4%	3.332	(2.805, 3.869)	3.385	(2.896, 3.871)
Low	No ABV; No ABV	2.549	(2.070, 3.092)	2.510	(2.014, 3.055)
Super Low	0%; 0%	2.441	(1.918, 3.022)	2.347	(1.842, 2.926)
Super Low	4%; 1%	2.718	(2.181, 3.288)	2.576	(1.980, 3.209)
Super Low	6%; 2%	2.782	(2.307, 3.307)	2.705	(2.174, 3.263)
Super Low	8%; 3%	3.144	(2.590, 3.693)	3.154	(2.660, 3.670)
Super Low	10%; 4%	3.060	(2.538, 3.582)	3.521	(2.995, 4.054)
Super Low	No ABV; No ABV	2.830	(2.302, 3.400)	2.724	(2.199, 3.276)

Understanding of alcohol strength

Item 1 – level of perceived appropriateness for children to consume given drink

Table S8a. *Logistic regression model on level of perceived appropriateness for children to consume given drink in wine drinkers.*

Experimental Condition	Estimate	Std. Error	z value	Pr(>|z|)	Sig	Sig (global)
(Intercept)	2.708	0.422	6.423	<0.0001	***	TRUE
Low label	-1.982	0.477	-4.158	<0.0001	***	TRUE
Super Low label	-1.901	0.477	-3.984	0.0001	***	TRUE
ABV 0%	-3.059	0.472	-6.484	<0.0001	***	TRUE
ABV 4%	-1.703	0.480	-3.549	0.0004	***	TRUE
ABV 6%	-1.159	0.499	-2.323	0.0202	*	FALSE
ABV 8%	-1.776	0.481	-3.693	0.0002	***	TRUE
ABV 10%	-1.150	0.503	-2.284	0.0224	*	FALSE
Low:0%	1.039	0.579	1.793	0.073	.	FALSE
SuperLow:0%	1.461	0.568	2.572	0.0101	*	FALSE
Low:4%	2.501	0.593	4.219	<0.0001	***	TRUE
SuperLow:4%	1.754	0.575	3.048	0.0023	**	TRUE
Low:6%	1.576	0.599	2.633	0.0085	**	FALSE
SuperLow:6%	1.518	0.595	2.553	0.0107	*	FALSE
Low:8%	2.337	0.584	3.998	0.0001	***	TRUE
SuperLow:8%	2.278	0.587	3.879	0.0001	***	TRUE
Low:10%	1.684	0.603	2.790	0.0053	**	TRUE
SuperLow:10%	1.561	0.604	2.584	0.0098	**	FALSE

Note. Global significance level ($p < .05$) when correcting for multiple comparisons. Only effect estimates denoted with TRUE in the column Sig (global) are significant when correcting for multiple comparisons. The intercept (comparison group) is the experimental condition denoting a regular strength drink (labelled with No verbal descriptor and No %ABV).

Table S8b. *Logistic regression model on level of perceived appropriateness for children to consume given drink in beer drinkers.*

Experimental Condition	Estimate	Std. Error	z value	Pr(>|z|)	Sig	Sig (global)
(Intercept)	1.920	0.323	5.945	<0.0001	***	TRUE
Low label	-0.821	0.397	-2.068	0.0386	*	FALSE
Super Low label	-0.901	0.396	-2.277	0.0228	*	FALSE
ABV 0%	-2.546	0.392	-6.493	<0.0001	***	TRUE
ABV 1%	-1.101	0.390	-2.822	0.0048	**	TRUE
ABV 2%	-0.189	0.434	-0.436	0.6629		FALSE
ABV 3%	-0.226	0.422	-0.536	0.592		FALSE
ABV 4%	0.577	0.509	1.134	0.2566		FALSE
Low:0%	0.801	0.503	1.591	0.1115		FALSE
SuperLow:0%	0.897	0.503	1.785	0.0743	.	FALSE
Low:1%	0.988	0.510	1.937	0.0528	.	FALSE
SuperLow:1%	0.919	0.510	1.803	0.0714	.	FALSE
Low:2%	0.232	0.547	0.424	0.6717		FALSE
SuperLow:2%	-0.007	0.539	-0.013	0.9896		FALSE
Low:3%	0.528	0.548	0.962	0.3359		FALSE
SuperLow:3%	0.516	0.542	0.952	0.3413		FALSE
Low:4%	0.270	0.638	0.423	0.6721		FALSE
SuperLow:4%	-0.085	0.619	-0.138	0.8906		FALSE

Note. Global significance level ($p < .05$) when correcting for multiple comparisons. Only effect estimates denoted with TRUE in the column Sig (global) are significant when correcting for multiple comparisons. The intercept (comparison group) is the experimental condition denoting a regular strength drink (labelled with No verbal descriptor and No %ABV).

Table S9. *Proportion (95% CI) estimates across the 18 experimental conditions on level of perceived appropriateness for consumption by children in wine and beer drinkers*.

		Wine		Beer	
Verbal Descriptor	ABV [Wine; Beer]	Proportion (%)	95% CI	Proportion (%)	95% CI
No Verbal Descriptor	0%; 0%	41.304	(25.000, 56.743)	34.831	(19.101, 50.562)
No Verbal Descriptor	4%; 1%	73.196	(58.763, 86.598)	69.388	(54.082, 83.673)
No Verbal Descriptor	6%; 2%	82.474	(69.072, 93.814)	84.946	(72.043, 95.699)
No Verbal Descriptor	8%; 3%	71.739	(56.522, 85.870)	84.466	(72.816, 94.175)
No Verbal Descriptor	10%; 4%	82.609	(68.478, 93.478)	92.391	(81.522, 98.913)
No Verbal Descriptor	No ABV; No ABV	93.750	(84.375, 100.000)	87.209	(74.419, 96.512)
Low	0%; 0%	21.505	(8.602, 36.559)	34.375	(19.792, 50.212)
Low	4%; 1%	82.105	(68.421, 93.684)	72.826	(57.609, 86.957)
Low	6%; 2%	75.824	(60.440, 89.011)	75.789	(61.053, 88.421)
Low	8%; 3%	78.351	(63.918, 90.722)	80.220	(66.810, 92.308)
Low	10%; 4%	77.895	(64.211, 90.526)	87.500	(76.042, 96.875)
Low	No ABV; No ABV	67.391	(51.953, 82.609)	75.000	(60.000, 88.000)
Super Low	0%; 0%	31.183	(16.129, 46.237)	34.737	(20.000, 50.526)
Super Low	4%; 1%	70.213	(54.255, 85.106)	69.767	(53.252, 84.884)
Super Low	6%; 2%	76.238	(62.376, 89.109)	69.474	(53.684, 84.211)
Super Low	8%; 3%	78.723	(64.894, 91.489)	78.723	(63.830, 91.489)
Super Low	10%; 4%	77.174	(63.043, 90.217)	81.915	(68.085, 93.617)
Super Low	No ABV; No ABV	69.149	(54.039, 84.043)	73.469	(58.163, 86.735)

Understanding of alcohol strength

Item 2 – knowledge of drinks suitable for driving within the legal limit:

Table S10a. *Logistic regression model on knowledge of drinks suitable for driving within the legal limit in wine drinkers.*

Experimental Condition	Estimate	Std. Error	z value	Pr(>|z|)	Sig	Sig (global)
(Intercept)	2.708	0.422	6.423	<0.0001	***	TRUE
Low label	0.383	0.663	0.578	0.5633		FALSE
Super Low label	1.121	0.830	1.350	0.1769		FALSE
ABV 0%	17.858	1848.519	0.010	0.9923		FALSE
ABV 4%	1.153	0.830	1.389	0.1647		FALSE
ABV 6%	0.438	0.662	0.662	0.5081		FALSE
ABV 8%	0.383	0.663	0.578	0.5633		FALSE
ABV 10%	0.148	0.624	0.238	0.812		FALSE
Low:0%	-0.383	2607.164	0.000	0.9999		FALSE
SuperLow:0%	-1.121	2607.164	0.000	0.9997		FALSE
Low:4%	16.322	1819.098	0.009	0.9928		FALSE
SuperLow:4%	15.585	1828.748	0.009	0.9932		FALSE
Low:6%	0.266	1.101	0.242	0.8089		FALSE
SuperLow:6%	-0.365	1.208	-0.302	0.7626		FALSE
Low:8%	1.090	1.308	0.834	0.4045		FALSE
SuperLow:8%	-1.526	1.062	-1.437	0.1508		FALSE
Low:10%	1.304	1.289	1.012	0.3117		FALSE
SuperLow:10%	-1.314	1.038	-1.266	0.2056		FALSE

Note. Global significance level ($p < .05$) when correcting for multiple comparisons. Only effect estimates denoted with TRUE in the column Sig (global) are significant when correcting for multiple comparisons. The intercept (comparison group) is the experimental condition denoting a regular strength drink (labelled with No verbal descriptor and No %ABV).

Table S10b. *Logistic regression model on knowledge of drinks suitable for driving within the legal limit in beer drinkers.*

Experimental Condition	Estimate	Std. Error	z value	Pr(>|z|)	Sig	Sig (global)
(Intercept)	2.277	0.371	6.134	<0.0001	***	TRUE
Low label	0.036	0.510	0.071	0.9431		FALSE
Super Low label	2.297	1.072	2.144	0.032	*	FALSE
ABV 0%	18.289	1879.416	0.010	0.9922		FALSE
ABV 1%	18.289	1791.038	0.010	0.9919		FALSE
ABV 2%	1.124	0.694	1.618	0.1056		FALSE
ABV 3%	0.932	0.631	1.477	0.1397		FALSE
ABV 4%	1.529	0.806	1.899	0.0576	.	FALSE
Low:0%	-0.036	2608.994	0.000	1		FALSE
SuperLow:0%	-2.297	2615.592	-0.001	0.9993		FALSE
Low:1%	-0.036	2573.876	0.000	1		FALSE
SuperLow:1%	-2.297	2619.778	-0.001	0.9993		FALSE
Low:2%	1.106	1.271	0.870	0.3843		FALSE
SuperLow:2%	-1.155	1.582	-0.730	0.4652		FALSE
Low:3%	0.550	1.016	0.542	0.5879		FALSE
SuperLow:3%	-3.131	1.243	-2.519	0.0118	*	FALSE
Low:4%	-1.574	0.945	-1.665	0.0958	.	FALSE
SuperLow:4%	-3.859	1.335	-2.890	0.0038	**	FALSE

Note. Global significance level ($p < .05$) when correcting for multiple comparisons. Only effect estimates denoted with TRUE in the column Sig (global) are significant when correcting for multiple comparisons. The intercept (comparison group) is the experimental condition denoting a regular strength drink (labelled with No verbal descriptor and No %ABV).

Table S11. *Proportion (95% CI) estimates across the 18 experimental conditions on knowledge of drinks suitable for driving within the legal limit in wine and beer drinkers*.

		Wine		Beer	
Verbal Descriptor	ABV [Wine; Beer]	Proportion (%)	95% CI	Proportion (%)	95% CI
No Verbal Descriptor	0%; 0%	100.000	(100.000, 100.000)	100.000	(100.000, 100.000)
No Verbal Descriptor	4%; 1%	97.938	(91.753, 100.000)	100.000	(100.000, 100.000)
No Verbal Descriptor	6%; 2%	95.876	(88.660, 100.000)	96.774	(89.247, 100.000)
No Verbal Descriptor	8%; 3%	95.652	(88.043, 100.000)	96.117	(88.350, 100.000)
No Verbal Descriptor	10%; 4%	94.565	(85.870, 100.000)	97.826	(91.304, 100.000)
No Verbal Descriptor	No ABV; No ABV	93.750	(84.375, 100.000)	90.698	(80.233, 98.837)
Low	0%; 0%	100.000	(100.000, 100.000)	100.000	(100.000, 100.000)
Low	4%; 1%	100.000	(100.000, 100.000)	100.000	(100.000, 100.000)
Low	6%; 2%	97.802	(91.209, 100.000)	98.947	(93.684, 100.000)
Low	8%; 3%	98.969	(94.845, 100.000)	97.802	(91.209, 100.000)
Low	10%; 4%	98.947	(94.737, 100.000)	90.625	(80.208, 98.958)
Low	No ABV; No ABV	95.652	(86.957, 100.000)	91.000	(81.000, 98.000)
Super Low	0%; 0%	100.000	(100.000, 100.000)	100.000	(100.000, 100.000)
Super Low	4%; 1%	100.000	(100.000, 100.000)	100.000	(100.000, 100.000)
Super Low	6%; 2%	98.020	(92.079, 100.000)	98.947	(94.737, 100.000)
Super Low	8%; 3%	93.617	(84.043, 100.000)	91.489	(81.915, 98.936)
Super Low	10%; 4%	93.478	(83.696, 100.000)	90.426	(79.787, 98.936)
Super Low	No ABV; No ABV	97.872	(91.489, 100.000)	98.980	(94.898, 100.000)

Understanding of alcohol strength

Item 3 – understanding of units contained in a small glass (125ml)/half pint of a given drink:

Table S12a. *Logistic regression model on understanding of units contained in a small glass (125ml) of wine in wine drinkers.*

Experimental Condition	Estimate	Std. Error	z value	Pr(>|z|)	Sig	Sig (global)
(Intercept)	-0.042	0.204	-0.204	0.8383		FALSE
Low label	0.438	0.295	1.486	0.1372		FALSE
Super Low label	1.552	0.337	4.608	<0.0001	***	TRUE
ABV 0%	18.608	680.032	0.027	0.9782		FALSE
ABV 4%	3.188	0.550	5.797	<0.0001	***	TRUE
ABV 6%	1.664	0.341	4.873	<0.0001	***	TRUE
ABV 8%	0.670	0.299	2.239	0.0251	*	FALSE
ABV 10%	0.920	0.307	3.000	0.0027	**	TRUE
Low:0%	-0.438	959.122	0.000	0.9996		FALSE
SuperLow:0%	-1.552	959.122	-0.002	0.9987		FALSE
Low:4%	-1.327	0.686	-1.935	0.053	.	FALSE
SuperLow:4%	-2.570	0.697	-3.686	0.0002	***	TRUE
Low:6%	-0.589	0.484	-1.218	0.2234		FALSE
SuperLow:6%	-2.115	0.490	-4.315	<0.0001	***	TRUE
Low:8%	-0.964	0.420	-2.296	0.0217	*	FALSE
SuperLow:8%	-2.053	0.452	-4.544	<0.0001	***	TRUE
Low:10%	-1.041	0.427	-2.439	0.0147	*	FALSE
SuperLow:10%	-2.605	0.458	-5.689	<0.0001	***	TRUE

Note. Global significance level ($p < .05$) when correcting for multiple comparisons. Only effect estimates denoted with TRUE in the column Sig (global) are significant when correcting for multiple comparisons. The intercept (comparison group) is the experimental condition denoting a regular strength drink (labelled with No verbal descriptor and No %ABV).

Table S12b. *Logistic regression model on understanding of units contained in a half pint of beer in beer drinkers.*

Experimental Condition	Estimate	Std. Error	z value	Pr(>|z|)	Sig	Sig (global)
(Intercept)	0.573	0.225	2.553	0.0107	*	FALSE
Low label	-0.291	0.302	-0.965	0.3345		FALSE
Super Low label	0.918	0.344	2.668	0.0076	**	FALSE
ABV 0%	17.993	691.398	0.026	0.9792		FALSE
ABV 1%	1.718	0.416	4.133	<0.0001	***	TRUE
ABV 2%	0.924	0.350	2.641	0.0083	**	FALSE
ABV 3%	0.005	0.304	0.018	0.9859		FALSE
ABV 4%	-0.086	0.311	-0.276	0.7828		FALSE
Low:0%	0.291	959.795	0.000	0.9998		FALSE
SuperLow:0%	-0.918	962.222	-0.001	0.9992		FALSE
Low:1%	-0.103	0.556	-0.185	0.8534		FALSE
SuperLow:1%	-1.391	0.581	-2.393	0.0167	*	FALSE
Low:2%	0.180	0.479	0.377	0.7064		FALSE
SuperLow:2%	-1.331	0.496	-2.683	0.0073	**	FALSE
Low:3%	0.229	0.425	0.540	0.589		FALSE
SuperLow:3%	-1.153	0.452	-2.550	0.0108	*	FALSE
Low:4%	-0.071	0.423	-0.168	0.8667		FALSE
SuperLow:4%	-1.449	0.455	-3.182	0.0015	**	TRUE

Note. Global significance level ($p < .05$) when correcting for multiple comparisons. Only effect estimates denoted with TRUE in the column Sig (global) are significant when correcting for multiple comparisons. The intercept (comparison group) is the experimental condition denoting a regular strength drink (labelled with No verbal descriptor and No %ABV).

Table S13. *Proportion (95% CI) estimates across the 18 experimental conditions on understanding of units in a given drink in wine and beer drinkers*.

		Wine		Beer	
Verbal Descriptor	ABV [Wine; Beer]	Proportion (%)	95% CI	Proportion (%)	95% CI
No Verbal Descriptor	0%; 0%	100.000	(100.000, 100.000)	100.000	(100.000, 100.000)
No Verbal Descriptor	4%; 1%	95.876	(87.629, 100.000)	90.816	(80.612, 98.980)
No Verbal Descriptor	6%; 2%	83.505	(71.134, 93.814)	81.720	(67.742, 93.548)
No Verbal Descriptor	8%; 3%	65.217	(48.913, 80.435)	64.078	(48.544, 78.641)
No Verbal Descriptor	10%; 4%	70.652	(55.214, 84.783)	61.957	(46.518, 78.261)
No Verbal Descriptor	No ABV; No ABV	48.958	(33.333, 64.583)	63.953	(47.674, 80.233)
Low	0%; 0%	100.000	(100.000, 100.000)	100.000	(100.000, 100.000)
Low	4%; 1%	90.526	(80.000, 98.947)	86.957	(75.000, 96.739)
Low	6%; 2%	81.319	(67.033, 93.407)	80.000	(66.316, 91.579)
Low	8%; 3%	52.577	(37.113, 69.072)	62.637	(46.154, 78.022)
Low	10%; 4%	56.842	(41.053, 72.632)	53.125	(37.500, 68.750)
Low	No ABV; No ABV	59.783	(42.391, 75.000)	57.000	(41.797, 73.000)
Super Low	0%; 0%	100.000	(100.000, 100.000)	100.000	(100.000, 100.000)
Super Low	4%; 1%	89.362	(77.660, 97.872)	86.047	(73.256, 96.512)
Super Low	6%; 2%	74.257	(59.406, 87.129)	74.737	(60.000, 87.582)
Super Low	8%; 3%	53.191	(37.018, 69.149)	58.511	(42.553, 74.468)
Super Low	10%; 4%	45.652	(29.348, 61.957)	48.936	(32.979, 64.894)
Super Low	No ABV; No ABV	81.915	(69.149, 92.553)	81.633	(68.367, 92.857)

Understanding of alcohol strength

Item 4 – understanding of the number of small glasses (125 ml)/half-pints of a given drink would match the alcohol contained in a small glass (125 ml)/half-pint of regular alcohol strength wine/beer:

Table S14a. *Logistic regression model on knowledge of understanding of the number of small glasses (125 ml) of a given drink would match the alcohol contained in a small glass (125 ml) of regular alcohol strength wine in wine drinkers.*

Experimental Condition	Estimate	Std. Error	z value	Pr(>|z|)	Sig	Sig (global)
(Intercept)	-0.379	0.208	-1.826	0.0678	.	FALSE
Low label	1.598	0.324	4.933	<0.0001	***	TRUE
Super Low	2.400	0.382	6.279	<0.0001	***	TRUE
ABV 0%	18.946	680.032	0.028	0.9778		FALSE
ABV 4%	2.245	0.363	6.180	<0.0001	***	TRUE
ABV 6%	1.437	0.312	4.613	<0.0001	***	TRUE
ABV 8%	0.467	0.295	1.584	0.1132		FALSE
ABV 10%	-0.396	0.306	-1.296	0.195		FALSE
Low:0%	-1.598	959.122	-0.002	0.9987		FALSE
SuperLow:0%	-2.400	959.122	-0.003	0.998		FALSE
Low:4%	-2.010	0.512	-3.925	0.0001	***	TRUE
SuperLow:4%	-2.605	0.561	-4.646	<0.0001	***	TRUE
Low:6%	-1.111	0.484	-2.293	0.0219	*	FALSE
SuperLow:6%	-2.060	0.512	-4.021	0.0001	***	TRUE
Low:8%	-1.022	0.441	-2.319	0.0204	*	FALSE
SuperLow:8%	-1.579	0.492	-3.211	0.0013	**	TRUE
Low:10%	-0.801	0.444	-1.803	0.0715	.	FALSE
SuperLow:10%	-1.362	0.491	-2.776	0.0055	**	FALSE

Note. Global significance level ($p < .05$) when correcting for multiple comparisons. Only effect estimates denoted with TRUE in the column Sig (global) are significant when correcting for multiple comparisons. The intercept (comparison group) is the experimental condition denoting a regular strength drink (labelled with No verbal descriptor and No %ABV).

Table S14b. *Logistic regression model on knowledge of number of half-pints of a given drink would match the alcohol contained in a half-pint of regular alcohol strength beer in beer drinkers.*

Experimental Condition	Estimate	Std. Error	z value	Pr(>|z|)	Sig	Sig (global)
(Intercept)	-0.573	0.225	-2.553	0.0107	*	FALSE
Low label	1.672	0.322	5.190	<0.0001	***	TRUE
Super Low	2.543	0.381	6.668	<0.0001	***	TRUE
ABV 0%	19.139	691.398	0.028	0.9779		FALSE
ABV 1%	3.303	0.477	6.919	<0.0001	***	TRUE
ABV 2%	2.807	0.416	6.740	<0.0001	***	TRUE
ABV 3%	1.069	0.303	3.529	0.0004	***	TRUE
ABV 4%	0.086	0.311	0.276	0.7828		FALSE
Low:0%	-1.672	959.795	-0.002	0.9986		FALSE
SuperLow:0%	-2.543	962.222	-0.003	0.9979		FALSE
Low:1%	-1.546	0.702	-2.202	0.0277	*	FALSE
SuperLow:1%	-2.488	0.732	-3.400	0.0007	***	TRUE
Low:2%	-2.382	0.546	-4.360	<0.0001	***	TRUE
SuperLow:2%	-3.021	0.593	-5.090	<0.0001	***	TRUE
Low:3%	-1.356	0.443	-3.059	0.0022	**	TRUE
SuperLow:3%	-2.377	0.484	-4.913	<0.0001	***	TRUE
Low:4%	-1.309	0.438	-2.991	0.0028	**	TRUE
SuperLow:4%	-2.013	0.484	-4.160	<0.0001	***	TRUE

Note. Global significance level ($p < .05$) when correcting for multiple comparisons. Only effect estimates denoted with TRUE in the column Sig (global) are significant when correcting for multiple comparisons. The intercept (comparison group) is the experimental condition denoting a regular strength drink (labelled with No verbal descriptor and No %ABV).

Table S15. *Proportion (95% CI) estimates across the 18 experimental conditions on number of small glasses (125 ml)/half-pints of a given drink matching the alcohol contained in a small glass (125 ml)/half-pint of regular alcohol strength in wine and beer drinkers*.

		Wine		Beer	
Verbal Descriptor	ABV [Wine; Beer]	Proportion (%)	95% CI	Proportion (%)	95% CI
No Verbal Descriptor	0%; 0%	100.000	(100.000, 100.000)	100.000	(100.000, 100.000)
No Verbal Descriptor	4%; 1%	86.598	(74.227, 96.086)	93.878	(84.694, 100.000)
No Verbal Descriptor	6%; 2%	74.227	(59.794, 87.629)	90.323	(79.570, 98.925)
No Verbal Descriptor	8%; 3%	52.174	(35.870, 68.478)	62.136	(46.602, 77.670)
No Verbal Descriptor	10%; 4%	31.522	(16.304, 46.739)	38.043	(21.739, 53.482)
No Verbal Descriptor	No ABV; No ABV	40.625	(25.000, 56.250)	36.047	(19.767, 52.326)
Low	0%; 0%	100.000	(100.000, 100.000)	100.000	(100.000, 100.000)
Low	4%; 1%	81.053	(68.421, 92.632)	94.565	(85.870, 100.000)
Low	6%; 2%	82.418	(69.231, 93.407)	82.105	(68.421, 93.684)
Low	8%; 3%	65.979	(50.515, 81.443)	69.231	(53.846, 83.516)
Low	10%; 4%	50.526	(34.523, 66.316)	46.875	(31.250, 62.500)
Low	No ABV; No ABV	77.174	(61.957, 90.217)	75.000	(60.000, 88.000)
Super Low	0%; 0%	100.000	(100.000, 100.000)	100.000	(100.000, 100.000)
Super Low	4%; 1%	84.043	(70.213, 94.681)	94.186	(84.884, 100.000)
Super Low	6%; 2%	80.198	(67.327, 92.079)	85.263	(72.632, 95.789)
Super Low	8%; 3%	71.277	(56.383, 85.106)	65.957	(50.000, 80.851)
Super Low	10%; 4%	56.522	(40.217, 72.826)	51.064	(35.106, 67.021)
Super Low	No ABV; No ABV	88.298	(76.596, 96.809)	87.755	(76.531, 96.939)

Calorie content estimation

Table S16a. *Logistic regression model on calorie estimation in wine drinkers.*

Experimental Condition	Estimate	Std. Error	z value	Pr(>|z|)	Sig	Sig (global)
(Intercept)	0.837	0.222	3.767	0.0002	***	TRUE
Low label	0.381	0.333	1.142	0.2533		FALSE
Super Low label	1.538	0.431	3.565	0.0004	***	TRUE
ABV 0%	1.514	0.432	3.508	0.0005	***	TRUE
ABV 4%	1.219	0.390	3.127	0.0018	**	TRUE
ABV 6%	1.028	0.372	2.766	0.0057	**	FALSE
ABV 8%	0.967	0.373	2.594	0.0095	**	FALSE
ABV 10%	0.576	0.344	1.674	0.0941	.	FALSE
Low:0%	-0.369	0.620	-0.595	0.552		FALSE
SuperLow:0%	-1.880	0.653	-2.880	0.004	**	FALSE
Low:4%	0.453	0.652	0.695	0.4869		FALSE
SuperLow:4%	-1.219	0.652	-1.870	0.0616	.	FALSE
Low:6%	-0.542	0.533	-1.016	0.3095		FALSE
SuperLow:6%	-1.491	0.603	-2.474	0.0134	*	FALSE
Low:8%	-0.563	0.525	-1.073	0.2831		FALSE
SuperLow:8%	-2.034	0.582	-3.492	0.0005	***	TRUE
Low:10%	-0.271	0.502	-0.540	0.5892		FALSE
SuperLow:10%	-1.670	0.565	-2.957	0.0031	**	TRUE

Note. Global significance level ($p < .05$) when correcting for multiple comparisons. Only effect estimates denoted with TRUE in the column Sig (global) are significant when correcting for multiple comparisons. The intercept (comparison group) is the experimental condition denoting a regular strength drink (labelled with No verbal descriptor and No %ABV).

Table S16b. *Logistic regression model on calorie estimation in beer drinkers.*

Experimental Condition	Estimate	Std. Error	z value	Pr(>|z|)	Sig	Sig (global)
(Intercept)	0.836	0.235	3.562	0.0004	***	TRUE
Low label	0.489	0.340	1.438	0.1503		FALSE
Super Low label	0.131	0.326	0.401	0.6887		FALSE
ABV 0%	1.791	0.484	3.703	0.0002	***	TRUE
ABV 1%	0.725	0.355	2.040	0.0413	*	FALSE
ABV 2%	0.894	0.373	2.396	0.0166	*	FALSE
ABV 3%	0.650	0.346	1.879	0.0602	.	FALSE
ABV 4%	0.150	0.332	0.451	0.6521		FALSE
Low:0%	-1.506	0.608	-2.479	0.0132	*	FALSE
SuperLow:0%	-1.161	0.600	-1.934	0.0531	.	FALSE
Low:1%	-0.414	0.516	-0.803	0.4222		FALSE
SuperLow:1%	-0.498	0.493	-1.011	0.312		FALSE
Low:2%	-0.622	0.524	-1.187	0.2351		FALSE
SuperLow:2%	-0.337	0.512	-0.659	0.5097		FALSE
Low:3%	-0.575	0.499	-1.152	0.2494		FALSE
SuperLow:3%	-0.106	0.493	-0.216	0.829		FALSE
Low:4%	-0.431	0.474	-0.909	0.3633		FALSE
SuperLow:4%	0.011	0.468	0.023	0.9816		FALSE

Note. Global significance level (p < .05) when correcting for multiple comparisons. Only effect estimates denoted with TRUE in the column Sig (global) are significant when correcting for multiple comparisons. The intercept (comparison group) is the experimental condition denoting a regular strength drink (labelled with No verbal descriptor and No %ABV).

Table S17. *Proportion (95% CI) estimates across the 18 experimental conditions on calorie estimation in wine and beer drinkers*.

		Wine		Beer	
Verbal Descriptor	ABV [Wine; Beer]	Proportion (%)	95% CI	Proportion (%)	95% CI
No Verbal Descriptor	0%; 0%	91.304	(80.435, 98.913)	93.258	(83.146, 100.000)
No Verbal Descriptor	4%; 1%	88.660	(77.320, 97.938)	82.653	(70.408, 93.878)
No Verbal Descriptor	6%; 2%	86.598	(74.227, 96.907)	84.946	(72.043, 95.699)
No Verbal Descriptor	8%; 3%	85.870	(73.913, 96.739)	81.553	(68.932, 93.204)
No Verbal Descriptor	10%; 4%	80.435	(66.304, 92.391)	72.826	(57.609, 86.957)
No Verbal Descriptor	No ABV; No ABV	69.792	(54.167, 84.375)	69.767	(53.488, 84.884)
Low	0%; 0%	91.398	(80.645, 98.925)	83.333	(70.833, 93.750)
Low	4%; 1%	94.737	(86.316, 100.000)	83.696	(70.652, 94.565)
Low	6%; 2%	84.615	(71.429, 95.604)	83.158	(69.474, 94.737)
Low	8%; 3%	83.505	(71.134, 93.814)	80.220	(65.711, 92.308)
Low	10%; 4%	82.105	(68.421, 93.684)	73.958	(59.375, 87.500)
Low	No ABV; No ABV	77.174	(63.043, 90.217)	79.000	(66.000, 91.000)
Super Low	0%; 0%	88.172	(76.344, 96.993)	83.158	(69.474, 94.737)
Super Low	4%; 1%	91.489	(80.851, 98.936)	76.744	(61.628, 89.535)
Super Low	6%; 2%	87.129	(75.248, 96.040)	82.105	(68.421, 92.845)
Super Low	8%; 3%	78.723	(64.894, 91.489)	81.915	(68.085, 93.617)
Super Low	10%; 4%	78.261	(64.130, 90.217)	75.532	(60.638, 89.362)
Super Low	No ABV; No ABV	91.489	(80.851, 98.936)	72.449	(58.163, 85.714)
